# A Network-Guided Genetic Approach to Identify Novel Regulators of Adventitious Root Formation in *Arabidopsis thaliana*

**DOI:** 10.3389/fpls.2019.00461

**Published:** 2019-04-12

**Authors:** Sergio Ibáñez, Helena Ruiz-Cano, María Á. Fernández, Ana Belén Sánchez-García, Joan Villanova, José Luis Micol, José Manuel Pérez-Pérez

**Affiliations:** ^1^Instituto de Bioingeniería, Universidad Miguel Hernández de Elche, Alicante, Spain; ^2^Instituto de Biología Molecular y Celular de Plantas, Universitat Politècnica de València-Consejo Superior de Investigaciones Científicas, Valencia, Spain; ^3^IDAI Nature S.L., La Pobla de Vallbona, Spain

**Keywords:** adventitious rooting, callus formation, gibberellin, ribosome, auxin homeostasis, xylem differentiation

## Abstract

Adventitious roots (ARs) are formed *de novo* during post-embryonic development from non-root tissues, in processes that are highly dependent on environmental inputs. Whole root excision from young seedlings has been previously used as a model to study adventitious root formation in *Arabidopsis thaliana* hypocotyls. To identify novel regulators of adventitious root formation, we analyzed adventitious rooting in the hypocotyl after whole root excision in 112 T-DNA homozygous leaf mutants, which were selected based on the dynamic expression profiles of their annotated genes during hormone-induced and wound-induced tissue regeneration. Forty-seven T-DNA homozygous lines that displayed low rooting capacity as regards their wild-type background were dubbed as the *less adventitious roots (lars)* mutants. We identified eight lines with higher rooting capacity than their wild-type background that we named as the *more adventitious roots (mars)* mutants. A relatively large number of mutants in ribosomal protein-encoding genes displayed a significant reduction in adventitious root number in the hypocotyl after whole root excision. In addition, gene products related to gibberellin (GA) biosynthesis and signaling, auxin homeostasis, and xylem differentiation were confirmed to participate in adventitious root formation. Nearly all the studied mutants tested displayed similar rooting responses from excised whole leaves, which suggest that their affected genes participate in shared regulatory pathways required for *de novo* organ formation in different organs.

## Introduction

Adventitious roots (ARs) are formed *de novo* from non-root tissues (i.e., stems or leaves) after a stress episode, such as drought, flooding or physical damage (Steffens and Rasmussen, 2016). AR formation is a complex process influenced by a large set of exogenous and endogenous factors (Druege et al., 2018). In *Arabidopsis thaliana*, induction of ARs in the hypocotyl has been successfully achieved either by growing seedlings in the dark and transferring them to light conditions (Sorin et al., 2005) or upon whole root excision (Sukumar et al., 2013). In the hypocotyl, ARs originate from a cell layer reminiscent to the root pericycle and the newly initiated ARs share histological and developmental characteristics with lateral roots (Bellini et al., 2014; Verstraeten et al., 2014). A local increase in auxin-induced marker expression was observed shortly after whole root excision in a defined region of the hypocotyl with the highest expression localized to xylem pole pericycle cells. This expression pattern was dependent on ATP BINDING CASSETTE SUBFAMILY B 19 (ABCB19)-mediated polar auxin transport from the shoot (Sukumar et al., 2013). In addition, mutations of *PIN-FORMED1* (*PIN1*) produced fewer ARs on de-rooted hypocotyls, while the PIN6 auxin transporter behave as a negative regulator of AR formation in this organ (Simon et al., 2016). In most species, however, ARs originate from non-root tissues, such as the vascular cambium, in a process that requires cell dedifferentiation and presumably different regulatory pathways as the hypocotyl-derived ARs (Bellini et al., 2014; Verstraeten et al., 2014).

Recent work has uncovered some of the molecular mechanisms that regulate the development of ARs from the hypocotyl (Gutierrez et al., 2009, 2012; Pacurar et al., 2014) and from excised whole leaves (Chen et al., 2014; Bustillo-Avendaño et al., 2018). Downstream of canonical auxin signaling pathway (Salehin et al., 2015), AUXIN RESPONSE FACTOR 6 (ARF6) and ARF8 are positive regulators of light-induced adventitious rooting in the hypocotyl, while ARF17 is a negative regulator of this process (Gutierrez et al., 2009). The ARF6/8/17 transcription factor network regulates the expression of three *GRETCHEN HAGEN 3* (*GH3*) genes, encoding acyl acid amido synthases, that lead to a net increase in jasmonic acid (JA) conjugation, which has been proposed to negatively regulate AR formation downstream of auxin (Gutierrez et al., 2012). Additional auxin signal transduction components involved in AR formation have been identified from a suppressor screen of the auxin overproducing *superroot2* (*sur2*) mutants, such as COP9 SIGNALOSOME SUBUNIT 4 (Pacurar et al., 2014, 2017), AUXIN RESPONSE 1 (AXR1), SHORT HYPOCOTYL 2 (SHY2), and RUB-CONJUGATING ENZIME 1 (RCE1), among others (Pacurar et al., 2014).

Based on the hypothesis that there are similar regulatory mechanisms in the formation of ARs and callus (Liu et al., 2014), the search for differentially induced genes in leaf explants and callus led to identification of *WOX11*, encoding a homeodomain transcription factor of the WUSCHEL HOMEOBOX (WOX) family (van der Graaff et al., 2009). In Arabidopsis leaf explants, *WOX11* directly responds to a wound-induced auxin maximum in and surrounding the procambium and acts redundantly with its homolog *WOX12* to upregulate *LATERAL ORGAN BOUNDARIES DOMAIN16* (*LBD16*) and *LBD29*, resulting in the first step of stem cell fate transition from procambial cells to root founder cells (Liu et al., 2014). Leaf explants displaying a constitutive overexpression of *WOX11* produced more ARs, while *wox11 wox12* explants or a dominant repressor mutant of *WOX11* produced fewer roots than the wild type (Liu et al., 2014). In turn, WOX11 and WOX12 activate WOX5 and WOX7 in dividing cells of the newly formed root primordia, while the subsequent WOX11and WOX12 expression quickly decreases in these cells (Liu et al., 2014; Hu and Xu, 2016). AR formation in leaf explants is also dependent on the endogenous basipetal transport system that concentrates the auxin generated in leaf blade mesophyll toward vascular cells near the cutting site (Liu et al., 2014; Chen et al., 2016). We recently proposed that an auxin-dependent switch in PIN3 polarization contributing to auxin-flow reversal is involved in maintaining high auxin levels in the vasculature near the cutting site during root regeneration (Bustillo-Avendaño et al., 2018). Factors primarily involved in lateral root formation, such as *CRANE* (also named as *IAA28*) and *SOLITARY ROOT* (also named as *IAA14*) are involved in rooting of leaves, suggesting the existence of partially overlapping auxin signaling modules during post-embryonic development (Bustillo-Avendaño et al., 2018).

Despite the remarkable advances in molecular-level understanding of the process of AR formation in Arabidopsis, not much is known about the downstream effectors of this complex response. Adventitious rooting requires activation of cell proliferation in root competent cells followed by founder cell specification in a subset of these cells, that they will be later committed to become a root (Bustillo-Avendaño et al., 2018). Based on the hypothesis that there are similar regulatory mechanisms in AR formation and other regenerative processes, such as callus formation (Lup et al., 2016), we selected a number of differentially expressed genes whose inactivation was previously known to affect leaf development (Wilson-Sánchez et al., 2014) to screen for mutants affected in wound-induced AR formation. Our results highlight novel regulation of ribosome function, gibberellin (GA) and auxin homeostasis that appears to be both complex and context specific.

## Materials and Methods

### Plant Materials and Growth Conditions

*Arabidopsis thaliana* wild-type accession Columbia-0 (Col-0) and confirmed T-DNA homozygous lines were obtained from the PhenoLeaf collection^1^ (Wilson-Sánchez et al., 2014). The following lines were used to isolate additional T-DNA homozygous mutants of the studied genes: N492755 (GK-967B07), N667578 (Salk_147826), N668393 (Salk_062900), N678155 (Salk_016729), and N840465 (Sail_896_G05) (Table 1). The *pDR5::GUS* (Ulmasov et al., 1997) line was used to investigate auxin response. Homozygous mutants of *DELLA* pentuple mutant (*dellaP*; Park et al., 2013), *gai-1* (Alabadí et al., 2008), *ga1-7* (Sun et al., 1992), *ga5-1* (Xu et al., 1995), and *ref2-1* (Hemm et al., 2003) were also used. Seedlings with T-DNA homozygous insertions in the studied genes were identified by sulfadiazine selection (N462401 and N492755) and PCR verification with T-DNA specific primers (the LBb1.3 primer for the Salk lines, the LB3 primer for the Sail lines, and the o8474 primer for the GABI-Kat lines) and a pair of gene-specific primers (Table 1). Genomic DNA isolation and genotyping of the T-DNA insertions were performed as described elsewhere (Pérez-Pérez et al., 2004).

**Table 1:** Oligonucleotides used in this study.

Gene	NASC ID (PhenoLeaf ID)	Stock/primer	Oligonucleotide sequences (5′→3′)	
At4g02780	N656319 (*m240*)	Salk_027931	TTGCCTACCAATTTTGAATGC	AATCCAAAACAAATGCATTGC
	N492755	GK_967B07	GCGGTTCCATACATTGTTC	CTTGTAAGCTTTAGCTCTTTC
At4g13770	N667207 (*m678*)	Salk_123405	TAGGAAGCAGAACAATGGTGG	GGCCTAAACTCATCAGGGTTC
At5g62190	N662659 (*m482*)	Salk_060686	TTTTCGTAAGACAAACCGCAG	CTTGTAATAAGGCAGCCATGG
	N668393	Salk_062900	TTGGGTTTTGCTTATTATGCG	AGAAGCAAGCGAAAAGGTCTC
	N678155	Salk_016729	TCGGTATTGTGAATCTCCTGC	ATATCAGGAATCAACCGAGCC
At5g64080	N655791 (*m232*)	Salk_103127	CATTTTGTTTCCTTTCACTTTC	TGTTGCTCCAAGTACTGCTCC
	N667578	Salk_147826	ATTTTTGTTTGGAAACCCCTG	TGGAGCAGTACTTGGAGCAAC
	N840465	Sail_896_G05	CTGTAGATGAATCGTGGAGGC	CGAACAGTCTACAGACGGAGC
T-DNA		LBb1.3	ATTTTGCCGATTTCGGAAC	
		LB3	TAGCATCTGAATTTCATAACCAATCTCGATACAC	
		o8474	ATAATAACGCTGCGGACATCTACATTTT	


**FIGURE 1:**
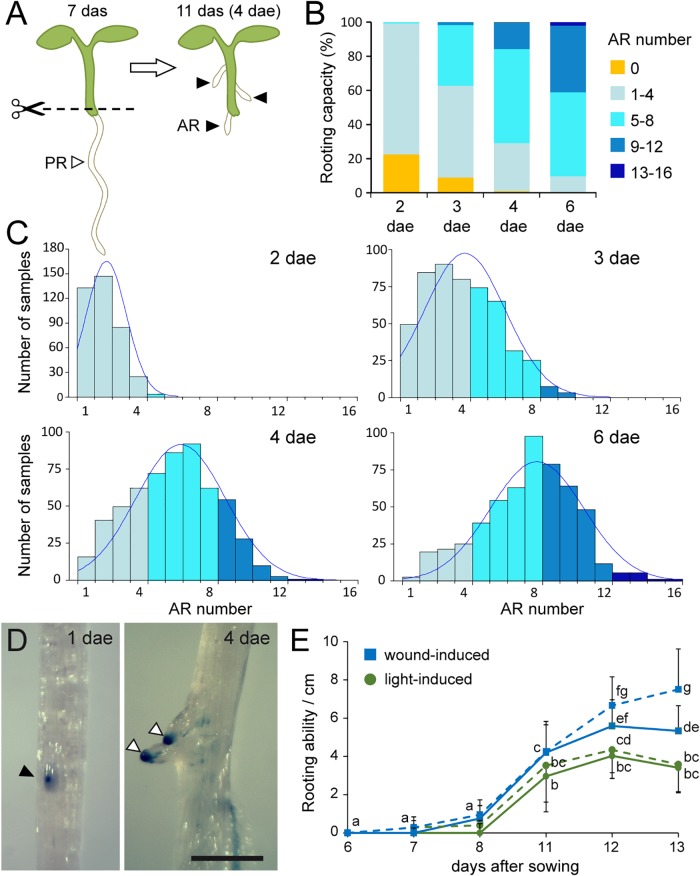
Whole root excision promotes adventitious root formation in Arabidopsis hypocotyls. **(A)** Schematic representation of the induction system used. Dotted lines indicate the cut site. AR, adventitious root; dae, days after excision; das, days after sowing; PR, primary root. **(B)** Multicolored barplots show frequencies of ARs from 2 to 6 dae. **(C)** Histograms for AR number in a large sample of Col-0 seedlings with overlay of theoretical normal distribution. **(D)** Representative images of GUS expression foci (black arrowhead) in the hypocotyl of *pDR5::GUS* lines. White arrowheads mark the emerged AR primordia. Scale bar: 1 mm. **(E)** Rooting ability of the hypocotyl estimated by the presence of AR primordia (continuous lines) or GUS expression foci (dotted lines) of *pDR5::GUS* lines. Different letters indicate significant differences (LSD; *p*-value < 0.01) at each data point.

Seeds were surfaced-sterilized in 2% (w/v) NaClO and rinsed with sterile water before being transferred to 120 × 120 × 10 mm Petri dishes containing 65 mL of one-half-Murashige and Skoog medium with 2% sucrose and 3 g L^-1^ Gelrite (Duchefa Biochemie, Netherlands). After 2 days of stratification at 4°C in darkness, plates were wrapped in aluminum foil and were transferred to an MLR-352-PE growth chamber (Panasonic, Japan) at 22 ± 1°C during 4 days in a nearly vertical position to induce hypocotyl elongation. Plates were unwrapped and grew during another 3 days with continuous light (50 μmol⋅m^-2^⋅s^-1^). The formation of ARs was then induced by removing the entire root system 1–2 mm above the hypocotyl-root junction with a sharp scalpel (Figure 1A). After whole root excision, seedlings were transferred to new Petri dishes containing growth media with 3% sucrose. The number of ARs in each hypocotyl was daily scored up to 6 days after excision (dae). Each Petri dish contained seedlings of two different lines and the Col-0 background (Eight seedlings per genotype). The experiment was performed in triplicate.

For assaying *de novo* root organogenesis in leaves, we followed the protocol described in Bustillo-Avendaño et al. (2018). Briefly, surface-sterilized seeds were sown in Petri dishes, and transferred to the growth chamber in horizontal position after 2 days of stratification at 4°C in darkness. 12 days after sowing (das), the first pair of leaves was excised across the junction of the petiole with the stem and the leaf explants, and they were transferred to new Petri dishes containing growth media with 3% sucrose. The number of ARs was scored up to 10 dae or for the number of days indicated in the corresponding experiment.

### Antibiotic Treatments

For antibiotic inhibition of ribosome function, leaf explants were incubated on growth medium supplemented with 30 μg ml^-1^ streptomycin that targets the small subunit of the chloroplast ribosomes.

### GUS Staining, Microscopic Observation, and Microphotography

For β-glucuronidase (GUS) staining, *pDR5::GUS* seedlings were incubated at 37°C for a minimum of 12 h in multi-well culture plates in the presence of the GUS staining solution as described in Pérez-Pérez et al. (2010). Leaf and hypocotyl samples were fixed in 96% ethanol for 48 h and washed with 0.1 M phosphate buffer (pH 6.8) before being transferred to clearing solution (80 g chloral hydrate and 30 mL distilled water) for chlorophyll removal. The area of proliferating vasculature was manually drawn from microscopic images using a Bamboo tablet (Wacom) and areas were measured with the software ImageJ (v1.50; National Institutes of Health). Bleached samples were mounted on slides using a mixture of 80 g chloral hydrate, 20 mL distilled water and 10 mL glycerol. Leaf pictures were obtained in a bright field Olympus AX70 microscope equipped with an Olympus PM-C35DX microphotography system (Olympus, Japan). Rosette, hypocotyl and leaf images were obtained with a SMZ-168-TL Stereo Zoom Microscope equipped with a Motic5+ digital camera (Motic, China).

### Heat Map Representation

We searched available gene expression data regarding several plant tissue regeneration experiments (Che et al., 2006; Sena et al., 2009; Sugimoto et al., 2010) available at the Arabidopsis eFP Browser within the Bio-Analytic Resource for Plant Biology (BAR) website^2^ (Winter et al., 2007). Gene expression data was retrieved for the 339 expressed genes with confirmed homozygous T-DNA insertions in the studied mutants of the PhenoLeaf collection (Wilson-Sánchez et al., 2014) that were available at the start of this project. In each experiment, we calibrated the expression value of the different conditions to its reference background and log2 transformed the output for outlier detection and convenient graphical representation. The standardized dataset obtained in this way (Supplementary Table S1) was processed using the pheatmap package of R version 3.3.2^3^. Euclidean distance matrixes between genes (rows) were calculated to build the dendrogram.

For a visual representation of the mutant phenotypes found, we built a data matrix containing normalized values for some of the estimated parameters (average, standard deviation, maximum, and minimum values) and a dendrogram was built with this dataset using Manhattan distances between mutant lines (rows).

### Statistical Analysis

Descriptive statistics (average, standard deviation, median, maximum, and minimum) were calculated by using the StatGraphics Centurion XV software (StatPoint Technologies, United States) and SPSS 21.0.0 (SPSS Inc., United States) programs. One-sample Kolmogorov–Smirnov tests were performed to analyze the goodness-of-fit between the distribution of the data and a theoretical normal distribution. To compare the data for a given variable, we performed multiple testing analyses with ANOVA *F*-test or Fisher’s LSD (Least Significant Differences) methods. For rooting capacity in excised leaves, χ^2^ test was performed to assay if there were differences in distribution frequency between lines, analyzed two-by-two. Significant differences were collected with 5% level of significance (*p*-value < 0.05), unless otherwise indicated.

## Results

### Adventitious Root Formation in Hypocotyls After Whole Root Excision

Whole root excision from young seedlings was previously used as a model to study AR formation in *A. thaliana* hypocotyls (Correa et al., 2012; Sukumar et al., 2013). We removed the entire root system 1–2 mm above the hypocotyl-root junction of 7 days-old seedlings and the number of ARs was visually scored between 1 and 6 (dae; Figure 1A). We characterized AR formation in the Col-0 accession, which has been used as a background reference for the Salk Unimutant and GABI-Kat collections of sequence-indexed T-DNA lines (Li et al., 2007; O’Malley and Ecker, 2010). As early as 2 dae, 76.7% of root-excised hypocotyls developed between one and four ARs, whereas no sign of AR formation was found for the remaining ones (Figure 1B). Interestingly, the number of ARs per hypocotyl increased significantly over time. At 6 dae, all root-excised hypocotyls developed at least one AR while 49.3% of the Col-0 hypocotyls included between five to eight ARs and some hypocotyls (2.0%) developed >12 ARs (Figure 1B). Distribution of AR number in the studied Col-0 population best fitted a normal function at 4 and 6 dae (Figure 1C).

Root excision-induced ARs in the hypocotyl emerged from the pericycle and were dependent on local auxin transport; previous results suggested that the internal auxin distribution was modified by the root excision, which, in turn, and drove enhanced AR initiation in the hypocotyls (Sukumar et al., 2013). We wondered whether root excision either induced the specification of new AR primordia within the hypocotyl or acted as a trigger to initiate the development of already-present AR founder cells within the hypocotyl. Indeed, lateral root founder cells are early specified in the oscillation zone of the primary root and later activated in the elongation zone upon additional shoot-derived signals (Laskowski and Ten Tusscher, 2017; Du and Scheres, 2018). To estimate the internal rooting ability of the hypocotyl and its eventual modification by the whole root excision, we quantified the number of foci (i.e., discrete regions) expressing the *pDR5::GUS* marker (Figure 1D), used previously as a direct read-out for endogenous auxin response maxima (Ulmasov et al., 1997). We found that the number of *pDR5::GUS* foci increased in intact hypocotyls between 8 and 12 das to a maximum of 4.3 ± 1.5 foci cm^-1^ (Figure 1E) and 95.2% of them developed as functional ARs in the absence of whole root excision. On the other hand, the number of *pDR5::GUS* foci in root-excised hypocotyls increased significantly from 11 das onward to a maximum of 7.5 ± 2.1 foci cm^-1^ at 13 das, but only 71.1% of them emerged as functional ARs (Figure 1D).

### Candidate Regulators Selected From Gene Expression Data

**FIGURE 2:**
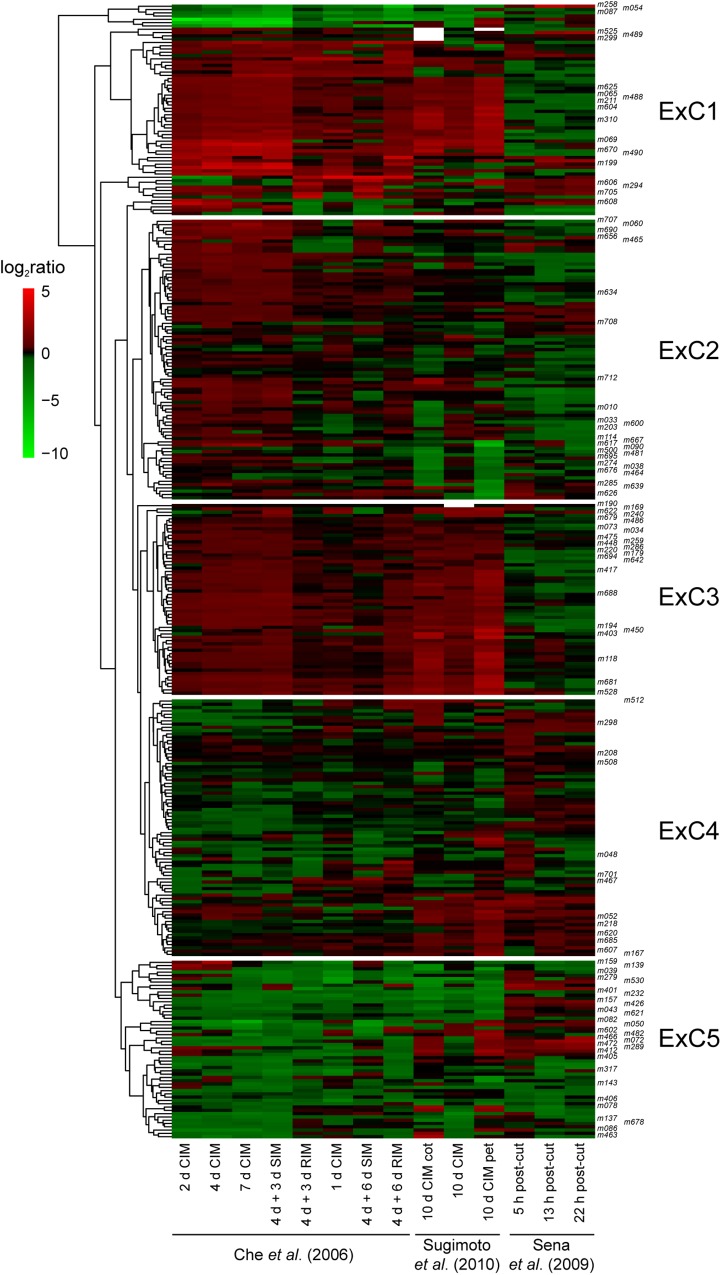
Gene expression clustering of 339 genes in the confirmed homozygous PhenoLeaf collection. Each row corresponds to a single gene, and the color scale corresponds to the log2 ratio of fold gene expression from green (downregulated genes) to red (upregulated genes) as regards the expression level of the mock treatment in each experiment, as indicated; white color specifies no expression data available. Euclidean distance matrix between genes was calculated to build the dendrogram. Five expression clusters, ExC1 to ExC5, were defined based on this dendrogram. CIM, callus induction medium; cot, cotyledon explants; pet, petal explants; RIM, root induction medium; SIM, shoot-induction medium. The studied mutants are indicated in the right corner.

To identify novel regulators of *de novo* root formation, we studied the annotated collection of T-DNA lines described previously (Wilson-Sánchez et al., 2014). 413 confirmed T-DNA homozygous lines that exhibit a leaf phenotype with full penetrance and constant expressivity were selected. To reduce the size of the screening population and to improve the frequency of the desired phenotypes, we prioritized candidate genes by using a network-guided genetic approach (Bassel et al., 2012; Ransbotyn et al., 2015). To this end, we gathered expression data for 339 of these genes from several Affymetrix microarray data sets related to hormone-induced and wound-induced tissue regeneration experiments (Che et al., 2006; Sena et al., 2009; Sugimoto et al., 2010; Supplementary Table S1), which were used to rank genes according to their expression profiles (Figure 2). Interestingly, while hormone-induced tissue regeneration followed an indirect morphogenesis pathway through callus formation a tissue with root primordium-like cell identity (Sugimoto et al., 2010), root tip regeneration proceeded through canonical WOX5, SCARECROW, and PLETHORA pathways required for root patterning and stem cell function (Sena et al., 2009; Lup et al., 2016). We reasoned that a positive regulator of hormone-induced tissue regeneration would increase its expression by the hormone treatment. In addition, such positive regulator will be expressed to a lesser extent during root tip regeneration as the reprogramming of this tissue proceeded by re-specification of lost cell identities in the absence of additional cell proliferation (Sena et al., 2009; Sena and Birnbaum, 2010). A contrasting expression profile was postulated for a negative regulator of hormone-induced tissue regeneration. By using these criteria, we selected 112 genes with dynamic expression profiles for further investigation (Figure 2).

### Search for Mutants Affected in Wound-Induced AR Formation in Hypocotyls

**FIGURE 3:**
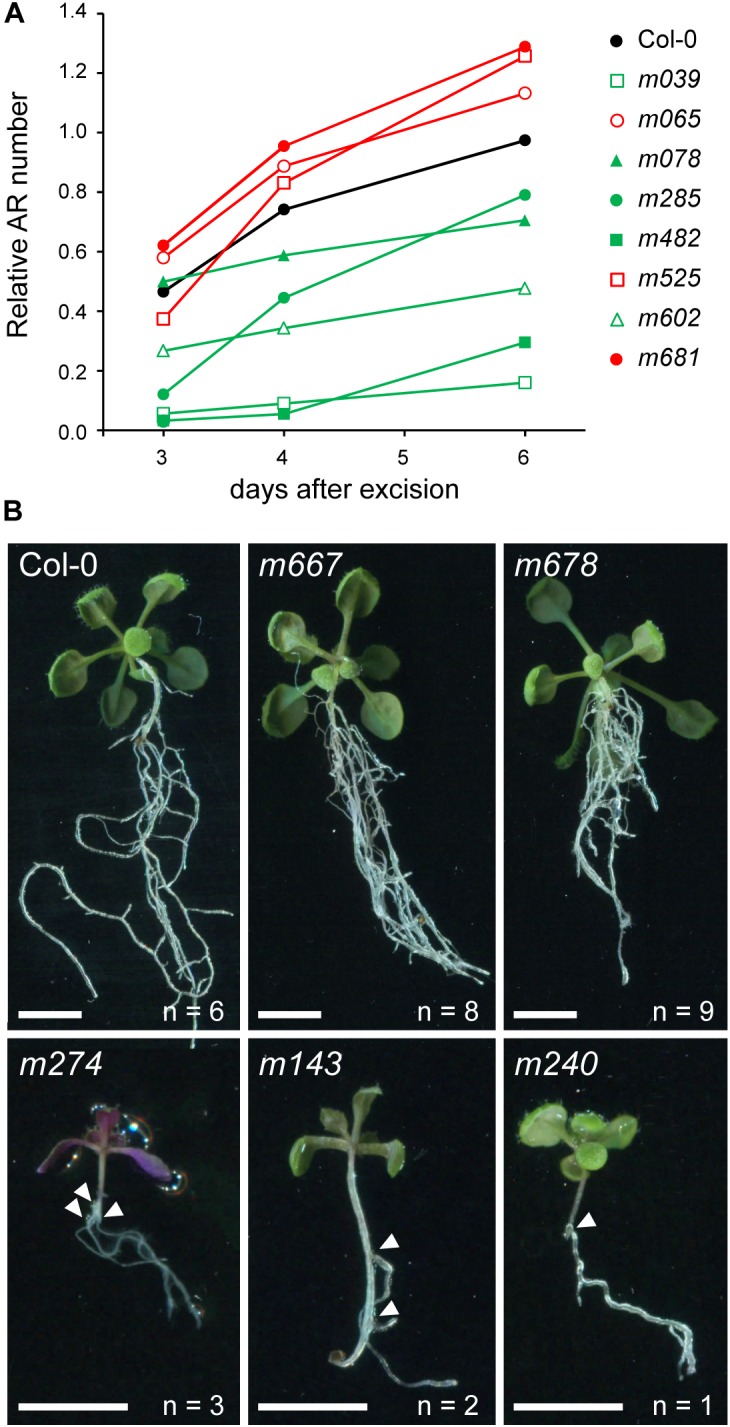
Some AR formation mutants in the hypocotyl after whole root excision. **(A)** Temporal analysis of AR formation in a representative sample of the studied genotypes displaying highly significant differences (green, with lower AR values; red, with higher AR values; LSD, *p*-value < 0.05 at both 3 and 4 dae. See Supplementary Table S2) as regards their Col-0 background (shown in black). **(B)** Representative images of rooted hypocotyls of some mutants with altered AR formation. Arrowheads indicate the site of AR initiation. Numbers beneath images indicate ARs in each plant. Scale bars: 5 mm.

We analyzed adventitious rooting in the hypocotyl after whole root excision of confirmed T-DNA homozygous lines in 112 selected genes (see section “Materials and Methods”). Mutant analysis was carried out in 11 consecutive sowings (S1 to S11) with Col-0 as a background reference. AR number in Col-0 ranged between 4.6 ± 1.5 (S6; *n* = 125) and 7.0 ± 2.2 (S7; *n* = 77) at 4 dae (Supplementary Figure S1). Normalized data for each mutant as regards Col-0 in the same sowing is shown in Supplementary Table S2. From the 112 mutants studied, 55 T-DNA homozygous lines (49.1%) showed a statistically significant difference (*p*-value < 0.05) in AR number as regards their Col-0 background in a minimum of two data points (Supplementary Figure S2 and Supplementary Table S2). Twenty-seven and 12 lines grouped together on the same phenotypic clusters, PhC5 and PhC6, respectively, and were dubbed as the *less adventitious roots* (*lars*) mutants (Supplementary Figure S2 and Supplementary Table S2). Eight remaining *lars* mutants were included in PhC4 and PhC3. Most *lars* mutants (e.g., *m039*, *m143*, *m240*, *m274, m482*, and *m602*) displayed low rooting capacity in the hypocotyl after whole root excision along the experiment (Figure 3A,B), indicating general defects in AR development. Other *lars* mutants either did not show clear defects in AR initiation but were delayed in subsequent AR emergence (e.g., *m078*) or were specifically affected at earlier time points (e.g., *m285*; Figure 3A,B).

We identified eight lines with enhanced rooting capacity as regards their Col-0 background (Figure 3A,B), five of them within the PhC1 cluster (Supplementary Figure S2 and Supplementary Table S2), that we named as the *more adventitious roots* (*mars*) mutants. Most of these *mars* mutants displayed a significant increase in AR number in all studied time points (e.g., *m065*, *m232*, *m667*, *m678*, and *m681*). Interestingly, the *m525* line only displayed a significant increase in AR number at later time points (Figure 3A).

### *De novo* Root Formation in Leaf Explants of Selected Mutants

**FIGURE 4:**
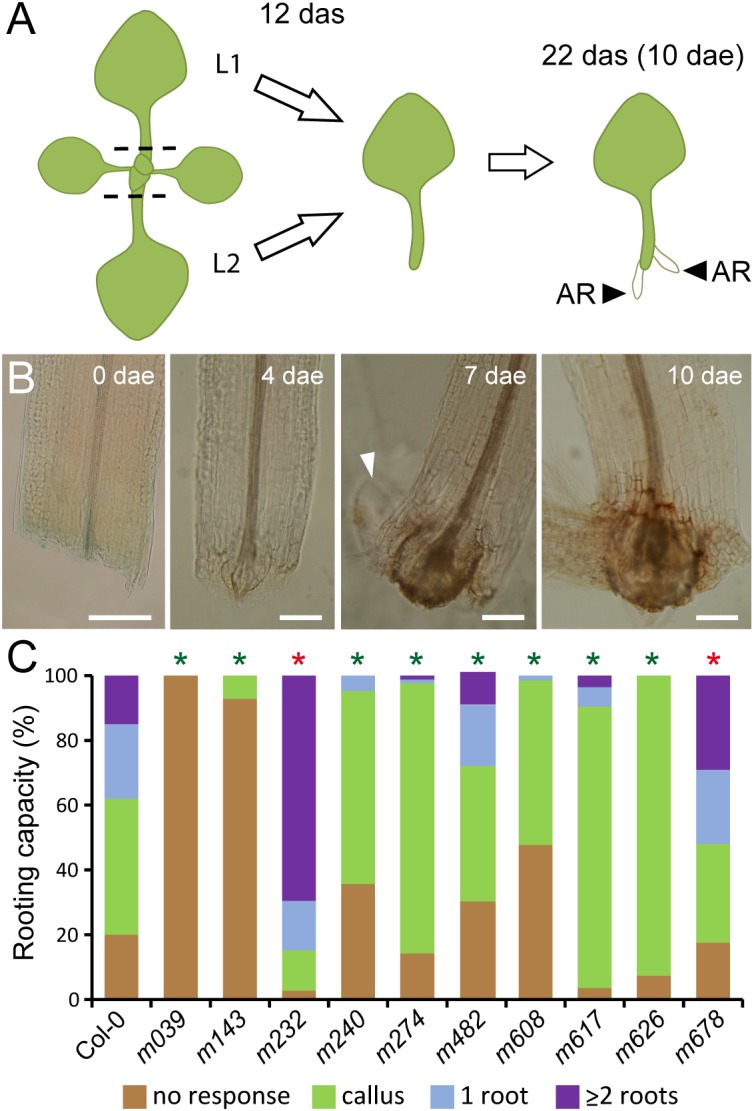
*De novo* root formation in selected mutants. **(A)** Schematic representation of *de novo* root formation in Arabidopsis whole leaf explants. Dashed lines indicate the cut site. AR: adventitious root; L1 and L2: first node and second node vegetative leaves, respectively. **(B)** Morphological changes at the petiole base during *de novo* root formation in the Col-0 background. Arrowhead indicates the emergence of a root primordium. Scale bars: 200 μm. **(C)** Rooting capacity in leaf explants of selected mutants at 10 dae. Asterisks indicate significant differences (LSD; *p*-value < 0.01) with the Col-0 background; those with lower AR values are shown in green and those with higher AR values are in red.

Adventitious roots might develop from different cell types depending on the tissue of origin (Bellini et al., 2014). Using the experimental set up described previously (Bustillo-Avendaño et al., 2018; Figure 4A,B), we analyzed the competence for *de novo* root formation in the petiole base of excised whole leaves of eight *lars* and two *mars* mutants (Figure 4C). On the one hand, some of the *lars* mutants studied (*m039*, *m143*, *m240*, and *m608*), showed a high percentage of leaf explants with no sign of vascular proliferation at the excision site which ultimately led to low AR responses (Figure 4C). On the other hand, the *lars* mutants *m274*, *m617*, and *m626* were able to activate vascular proliferation in most leaf explants although ARs were rarely initiated, indicating specific defects in the ectopic specification of root founder cells and/or root primordia initiation in these mutants (Figure 4C). *m232* and *m678* lines were selected as *mars* for their increased AR formation in the hypocotyl (Supplementary Figure S2) and effectively displayed increased percentages of leaf explants with more ARs at 10 dae than those of the Col-0 background (Figure 4C). These results confirmed that the mutants identified previously in the wound-induced hypocotyl AR formation screen also displayed *de novo* root organogenesis phenotypes in whole leaves, indicating the putative participation of the damaged genes in shared developmental programs required for AR formation in both hypocotyls and proximal petioles of excised leaves. Further analyses will be required to confirm if these gene functions are conserved in AR formation from other organs.

### Analysis of *lars* Mutants Reveals a Positive Role for Gibberellins and Ribosome Function in AR Formation

We previously estimated that the annotated T-DNA was responsible for the observed phenotype in ∼47% of the lines in the PhenoLeaf collection and that their average number of T-DNA insertions was 2.1 (Wilson-Sánchez et al., 2014). To confirm that the observed AR phenotype of studied lines was caused by the homozygosity at the annotated T-DNA insertion and not because of other, non-annotated, T-DNA insertions, we selected additional mutant alleles of the putative causal genes of two of the studied *lars* mutants, *m240*, and *m482* (Table 1).

**FIGURE 5:**
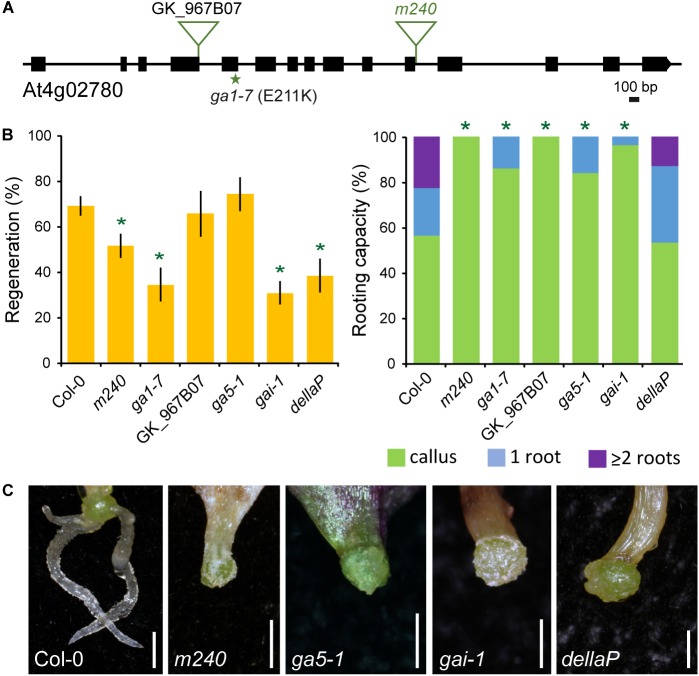
Functional analysis of *GA1* during AR formation. **(A)** Gene structure of At4g02780. Exons are represented by boxes and introns are depicted as lines. The studied mutant lines and annotated T-DNA insertion (triangles) lines are indicated. **(B)** Regeneration and rooting capacity in leaf explants of selected lines at 10 dae. Asterisks indicate significant differences (LSD; *p*-value < 0.01) with the Col-0 background; those with lower AR values are in green. **(C)** Representative images of whole leaf explants of the studied lines at 14 dae. Scale bars: 2 mm.

The *m240* mutant contained a homozygous T-DNA insertion at the 11th exon of the At4g02780 gene (Figure 5A), also named *GA REQUIRING 1*, which encodes the ENT-COPALYL DIPHOSPHATE SYNTHETASE 1 involved in a key step of GA biosynthesis (Michaels and Amasino, 1999). We analyzed rooting capacity in the petiole base of whole leaves of *ga1-7* mutants (Figure 5A) with a severe loss of GA1 function (Sun et al., 1992). *m240* and *ga1-7* leaf explants showed a strong reduction in their rooting capacity as regards their wild-type backgrounds, with most of the mutant explants showed lack or a severe delay of vascular proliferation (Figure 5B,C). In addition, we isolated additional T-DNA homozygous mutants within the *GA1* locus from the GK_967B07 line (Figure 5A) that also exhibited impaired rooting capacity in leaf explants (Figure 5B,C), supporting the correlation between defective AR formation and GA1 inactivation. To confirm the requirement for GA biosynthesis in AR formation, we studied the *ga5-1* mutant, which contains a loss-of-function in the GA 20-oxidase required for the later steps of GA biosynthesis (Xu et al., 1995). Consistently, *ga5-1* leaf explants showed a mild delay in AR formation (Figure 5B,C).

We wondered whether the effect of GAs in AR formation was dependent on canonical GA signaling pathway acting through DELLA repressors (Sun and Gubler, 2004). We analyzed AR formation in whole leaf explants of *gai-1*, bearing a deletion of the DELLA domain in GAI protein that renders this repressor constitutive and insensitive to GAs (Peng et al., 1997), and of the multiple mutant of all five *DELLA* genes (*dellaP*) that display constitutive GA responses (Park et al., 2013). Similar to GA-deficient mutants, *gai-1* leaf explants were partially defective on vascular proliferation and were consequently delayed in AR formation at 10 dae (Figure 5B,C). On the other hand, the *dellaP* mutants, with constitutive GA responses, also shown reduced regeneration percentage while the average number of ARs was not significantly different from those of the wild-type background (Figure 5B,C). Altogether, our results confirmed the requirement of GAs and tight regulation of their signaling through DELLA repressors to promote AR formation.

**FIGURE 6:**
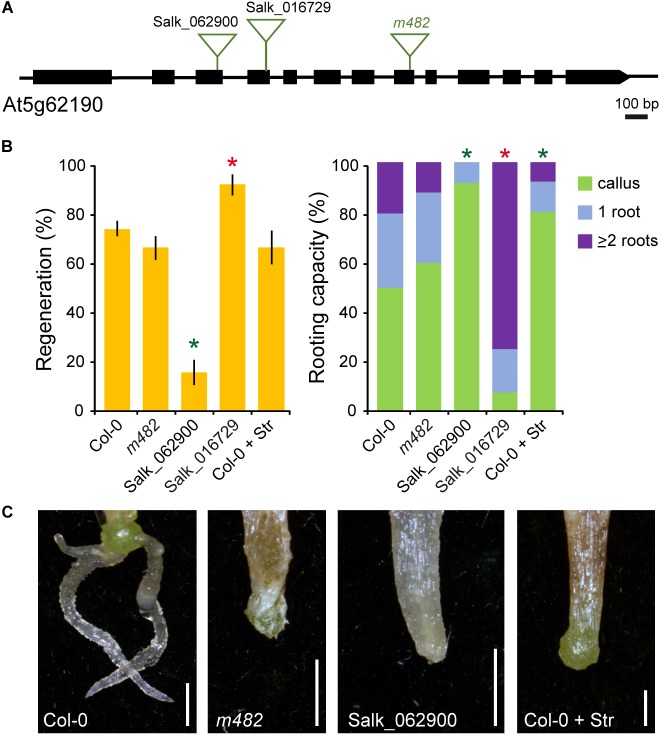
Functional analysis of *AtRH7/PRH75* during AR formation. **(A)** Gene structure of At5g62190. Exons are represented by boxes and introns are depicted as lines. The studied mutant lines and annotated T-DNA insertion (triangles) lines are indicated. **(B)** Regeneration and rooting capacity in leaf explants of selected lines and treatments at 10 dae. Asterisks indicate significant differences (LSD; *p*-value < 0.01) with the Col-0 background; those with lower AR values are in green and those with higher AR values in red. **(C)** Representative images of whole leaf explants of the studied lines and treatments at 14 dae. Str: 30 μg ml^-1^ streptomycin. Scale bars: 2 mm.

The *m482* mutant contained a homozygous T-DNA insertion at the 8th exon of the At5g62190 gene (Figure 6A), encoding the AtRH7/PRH75 DEAD-box RNA helicase involved in pre-rRNA processing which is active in regions undergoing cell division (Huang et al., 2016). We studied rooting capacity of *m482* (also named as *atrh7-2*) along with two additional T-DNA insertional lines of the *AtRH7/PRH75* locus (Figure 6A). All T-DNA homozygous mutants studied displayed a characteristic narrow leaf phenotype (Supplementary Figure S3) but only the Salk_062900 homozygotes displayed a significant lack of response during *de novo* root formation in the petiole base of whole leaves as compared with their Col-0 background (Figure 6B,C). Surprisingly, the Salk_016729 homozygotes displayed increased regeneration with a higher average of AR than in the wild-type background (Figure 6B,C). To confirm whether the defects in rRNA processing producing altered ribosome conformation might cause the observed AR phenotype of *AtRH7/PRH75* loss-of-function mutants, we incubated leaf explants on streptomycin that targets the small subunit of the chloroplast ribosomes and found a striking reduction of rooting capacity due to a delay in AR emergence (Figure 6B,C). Taken together, our results indicated that *AtRH7/PRH75* mutations might affect proper ribosome assembly, which is indeed required for AR development, an observation that requires further investigation.

### Organ-Dependent Auxin Homeostasis Influences AR Formation

**FIGURE 7:**
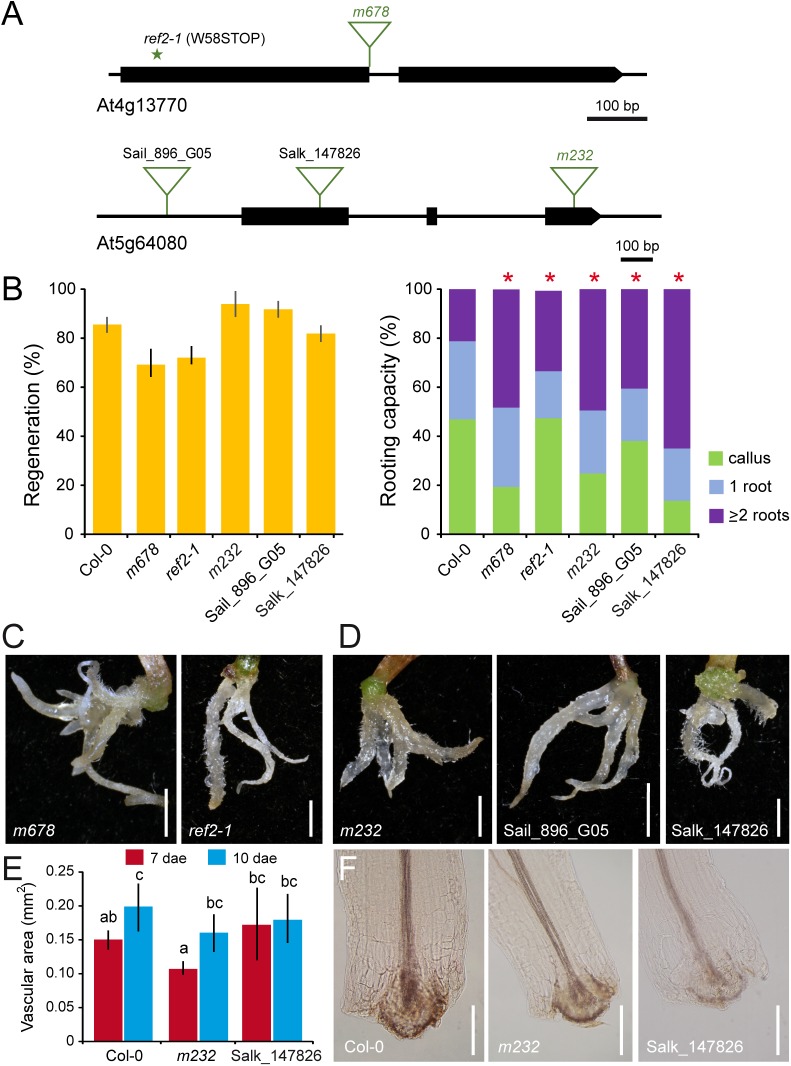
Functional analysis of *REF2* and *XYP1* during AR formation. **(A)** Gene structure of At4g13770 and At5g64080. Exons are represented by boxes and introns are depicted as lines. The studied annotated T-DNA insertion (triangles) lines are indicated. **(B)** Regeneration and rooting capacity in leaf explants of selected lines at 10 dae. Asterisks indicate significant differences (LSD; *p*-value < 0.01) with the Col-0 background; those with higher AR values are in red. **(C,D)** Representative images of whole leaf explants of the studied lines at 14 dae. Scale bars: 2 mm. **(E)** Area of the vascular region in the proximal petiole at 7 and 10 dae of selected *xyp1* mutants. Different letters indicate significant differences (LSD; *p*-value < 0.05). **(F)** Vascular proliferation on the proximal petiole at 7 dae of selected *xyp1* mutants. Scale bars: 200 μm.

The *m678* mutant was identified as a *mars* mutant in our wound-induced hypocotyl AR formation screen. *m678* carried a homozygous T-DNA insertion in *REDUCED EPIDERMAL FLUORESCENCE 2* (*REF2*), encoding the CYP83A1 enzyme involved in the initial conversion of aldoximes to thiohydroximates in tryptophan-independent glucosinolate biosynthesis pathway (Bak and Feyereisen, 2001; Nintemann et al., 2018). One additional loss-of-function allele of the *REF2* gene was tested for *de novo* root formation in the petiole base of whole leaves (Figure 7A), which also produced vegetative rosettes with small curled down leaves (Supplementary Figure S3). Similar to *m678* mutants, *ref2-1* homozygotes activated vascular proliferation in most leaf explants and a significantly higher number of ARs were produced from these tissues (Figure 7B,C).

### The Xylem Differentiating Factor XYP1 Is a Negative Regulator of AR Formation

The *mars* mutant *m232* was homozygous for a T-DNA insertion in the 3rd exon of the *XYLOGEN PROTEIN 1* (*XYP1*) locus (Figure 7A). XYP1 has been postulated as the xylogen factor for xylem differentiation (Motose et al., 2004). We identified homozygous mutants from two additional T-DNA insertional lines of the *XYP1* gene (Figure 7A). T-DNA homozygous mutants of the Sail_896_G05 line also developed more ARs than the Col-0 background (Figure 7B). Homozygous mutants in the Salk_147826 line showed a significant increase in rooting capacity of leaf explants at 10 dae as compared with those of Col-0 (Figure 7B,D). We wondered whether the increase number of ARs in leaf explants of *m232* and Salk_147826C homozygotes was caused by enhanced vasculature proliferation. The area of vascular proliferation on leaf explants at 7 and 10 dae was similar in these two mutants and the Col-0 background (Figure 7E,F), suggesting that the loss of XYP1 function enhanced post-embryonic root founder cell specification which ultimately lead to an increase in AR number.

## Discussion

We optimized a protocol to study wound-induced AR formation in *A. thaliana* hypocotyls, which is suitable for high-throughput mutant screens. Our results indicate that whole root-excision both triggered specification of new auxin-responsive (*pDR5::GUS*) foci and growth of already-specified auxin-responsive foci within the hypocotyl, leading to a significant increase in the number of ARs a few days after the excision. We found substantial variation in the rate of AR formation in the wild-type background among experiments, indicating an environmentally mediated regulation of this developmental response. Hypocotyl-derived ARs originated from xylem-pole pericycle cells in a process resembling lateral root initiation (Bellini et al., 2014; Verstraeten et al., 2014). In the current model for wound-induced AR formation in hypocotyls (Sukumar et al., 2013), root excision enhances polar auxin transport through the hypocotyl while auxin accumulation at the excision site drives localized specification of AR founder cells within the pericycle. In intact hypocotyls, polar auxin transport through the hypocotyl and toward active primary (and lateral) root meristems reduced auxin accumulation in hypocotyl pericycle cells, which, in turn, limits following AR emergence.

By combining gene profiling data and a systematic phenotypic screen, we identified a large number of leaf mutants with a pleiotropic phenotype on AR formation in hypocotyls after whole root excision. In our study, 47 (41.6%) and 8 (7.1%) of studied PhenoLeaf mutants displayed, respectively, significantly less and more wound-induced ARs in the hypocotyl than the Col-0 background. In most species, however, AR formation aroused from non-root tissues, such as the vascular cambium, in a process that requires cell dedifferentiation and presumably different regulatory pathways as hypocotyl-derived ARs (Druege et al., 2018). Hence, we assayed *de novo* root organogenesis in excised whole leaves (Bustillo-Avendaño et al., 2018) of selected AR mutants. Nearly all the studied mutants displayed similar AR responses in excised whole leaves too, which suggest that the genes affected in these mutants participated in shared regulatory pathways required for *de novo* organ formation from different organs.

We have identified in our screen a relatively large number of mutants in protein translation- and ribosomal protein-encoding genes that displayed a significant reduction in AR number in the hypocotyl after whole root excision (*n* = 11; 23.4% of the *lars* mutants studied). Some of them lacked specific ribosomal protein functions. An example of these were the *m274* mutant, which was homozygous for a T-DNA insertion in the At4g16720 gene encoding a ribosomal protein of the L23/L15e family (Carroll et al., 2008), and the *m285* mutant, which carried a T-DNA insertion in the *PIGGYBACK1* (*PGY1*) gene encoding the L10a ribosomal subunit (Pinon et al., 2008). Despite the known genetic redundancy between ribosomal protein-encoding genes (Carroll et al., 2008), some of their mutants exhibited rare developmental phenotypes (e.g., pointed leaves and auxin-related phenotypes) that suggest non-equivalent functions of ribosome paralogs for the translational regulation of specific target mRNAs (Horiguchi et al., 2012). Other *lars* mutants related to ribosome function were *m405*, *m482*, and *m602*. *m482*, and *m602* carried homozygous insertions in two genes, respectively, encoding the DEAD-box RNA helicases AtRH57 (Hsu et al., 2014) and AtRH7/PRH75 (Huang et al., 2016). Both genes were required for pre-rRNA processing (Liu and Imai, 2018). Interestingly, the *root initiation defective1-1* (*rid1-1*) mutant identified as a temperature sensitive allele of another DEAH-box RNA helicase-encoding gene showed reduced hormone-induced AR formation from hypocotyl explants (Konishi and Sugiyama, 2003; Ohtani et al., 2013). *m405* affects At3g09720, which encodes the large subunit of a GTPase required for maturation of the 60S ribosomal subunit and whose loss-of-function caused the alteration of auxin distribution, auxin response, and auxin transport, and consequently affecting multiple auxin-regulated developmental processes (Zhao et al., 2015). Our results are in agreement with a specific role for ribosomes as regulators of key patterning events in AR development. One possibility is that ribosome function influences the cell’s ability to undergo cell division during the early stages of AR formation (e.g., vasculature proliferation) or, alternatively, that certain set of genes involved in specific AR responses might require a particular ribosome conformation and therefore will be selectively regulated. Although the fact that mutant alleles of specific ribosomal protein-encoded genes caused a decrease in translational expression of particular auxin response factors (Rosado et al., 2012) favors the later hypothesis, its confirmation require further research.

Several lines of evidence support the hypothesis that active GAs are critical for primary root development through the control of root meristem size (Achard et al., 2009; Úbeda-Tomás et al., 2009). However, reports from various species suggest that GAs have an inhibitory effect on AR development (Niu et al., 2013; Mauriat et al., 2014). In hybrid aspen, transgenic plants with enhanced GA biosynthesis or signaling had significantly fewer ARs in stem cuttings, likely by the negative crosstalk of GAs with polar auxin transport (Mauriat et al., 2014). Analysis of GA-constitutive mutant *procera* (*pro*), a loss-of-function in a DELLA-like protein, also indicates that reduced levels or sensitivity to GA are associated with enhanced hormone-induced *in vitro* organogenesis in tomato (Lombardi-Crestana et al., 2012). However, we found that loss-of-function alleles of *GA1* and *GA5*, which are involved in key steps of GA biosynthesis (Xu et al., 1995; Michaels and Amasino, 1999), cause a significant decrease in AR numbers in both hypocotyl explants and excised leaves. In addition, we demonstrated that GA-related AR phenotypes were dependent on the growth-repressing DELLA function. Retarded growth of AR primordia in GA-deficient mutants was consistent with a positive role for GAs on both cell production and cell elongation in the root meristem (Achard et al., 2009; Úbeda-Tomás et al., 2009). In line with our results, intriguing results were found for GA function during AR formation in tobacco cuttings (Niu et al., 2013), which were interpreted as a consequence of GAs negatively regulating the early initiation step of AR formation but stimulating AR elongation. In all these examples, the relationship between GA biosynthesis and GA signaling appears to be both complex and context specific, which deserves further investigation.

The eight *mars* mutants that we identified might define negative regulators of AR formation. Among them, the *m667* mutants were homozygous for a T-DNA insertion in At2g45310 (*GAE4*), one of the six genes encoding UDP-D-glucuronate 4-epimerases involved in pectin biosynthesis (Molhoj et al., 2004; Usadel et al., 2004). Confirming the role for cell wall mechanics in AR initiation, the *atpme3-1* mutant, with low pectin methylesterase levels, also displayed a large increase (>30%) in the number of ARs emerging from the hypocotyl (Guenin et al., 2011). Indeed, fine-tuned crosstalk between microtubules (MTs), cell walls and auxin transport has been shown to be required for AR induction (Abu-Abied et al., 2015). In addition, MT perturbations caused a lack of PIN1 polarization and a loss of auxin maxima localization in the hypocotyl, which in turn lead to the formation of amorphous cell clusters and defective AR formation (Abu-Abied et al., 2015). In line with these results, the *m667* mutant might contain altered pectin levels in the wounded hypocotyl that interferes with PIN1 localization and auxin response during AR formation.

We identified two T-DNA insertions within the *XYP1* gene that caused a *mars* phenotype. *XYP1* is one of the genes encoding xylogen, an extracellular arabinogalactan protein that mediates local intercellular communication involved in xylem cell differentiation of *Zinnia elegans* cell cultures (Motose et al., 2004). According to previous studies, xylogen is secreted directionally from differentiating vascular cells, moves in the apoplast to the adjacent undifferentiated mesophyll cells and draws them into the pathway of vascular differentiation (Motose et al., 2004). In many species, the vascular cambium has been identified as the originating tissue for stem-derived ARs (Bellini et al., 2014; Druege et al., 2018). A definite population of indeterminate cambial initials that produce xylem mother cells inward and phloem mother cells outward from the cambium has been proposed to reside within the vascular cambium (Nieminen et al., 2015). It is therefore possible that reduced xylem differentiation in *xyp1* mutants will enlarge the number of these cambial initials allowing an auxin-mediated specification of a large population of AR founder cells and hence increasing the number of ARs formed in these mutants. Further studies using marker lines for AR founder cell specification (Bustillo-Avendaño et al., 2018) will help to confirm this hypothesis.

Another mutant with higher rooting capacity in wound-induced hypocotyls was *m678*, which is homozygous for a T-DNA insertion in *REF2*, encoding the CYP83A1 enzyme that catalyzes the conversion of aldoximes to thiohydroximates in the tryptophan-independent glucosinolate biosynthesis pathway (Bak and Feyereisen, 2001; Nintemann et al., 2018). Interestingly, the development of ARs from the hypocotyl is a well-known feature of the high-auxin phenotype of *superroot2-1* (*sur2-1*) mutant with a loss of function in CYP83B1, sharing 63% amino acid identity with CYP83A1 (Delarue et al., 1998; Barlier et al., 2000). The *ref2-1* and *sur2-1* mutants displayed reduced glucosinolate levels and increased levels of its precursors in leaves, suggesting a compensatory interplay between CYP83A1 and CYP83B1 in some organs (Hemm et al., 2003). The contrasting results found for *ref2* and *sur2-1* mutants in wound-induced AR formation in hypocotyls and whole leaves might be due to unequal genetic redundancy between *REF2* and *SUR2* (Briggs et al., 2006). Indeed, indole-3-acetaldoxime channeling into production of either indole-3-acetic acid (IAA) or glucosinolates is tightly controlled and could explain the high-auxin phenotypes of *ref2-1* and *sur2-1* mutants. Other glucosinolate biosynthesis mutants also have increased levels of IAA and therefore enhanced auxin responses, which indicates a direct interaction between the biosynthetic pathways of glucosinolates and auxin (Malka and Cheng, 2017).

We used a network-guided genetic approach on a well-characterized T-DNA mutant collection (PhenoLeaf) that allowed us to identify novel functions in AR development for genes involved in foreseen housekeeping functions. With the advent of new systems biology tools (Waese et al., 2017), candidate genes will be selected based on cell-specific expression, protein-protein and protein-DNA interaction, and high-throughput screening for AR phenotypes in multiple T-DNA insertional lines of each gene will be conducted.

## Author Contributions

JMP-P was responsible for conceptualization and supervision. SI, JV, and JMP-P were responsible for methodology. SI, HR-C, MÁF, ABS-G, and JV were involved in the investigation. SI and JMP-P performed the formal analysis and wrote the original draft. SI, JLM, and JMP-P were involved in the review and editing of the manuscript. JMP-P provided the funding acquisition. JLM provided the PhenoLeaf mutants studied here.

## Conflict of Interest Statement

The authors declare that the research was conducted in the absence of any commercial or financial relationships that could be construed as a potential conflict of interest.

## Footnotes

1 http://genetics.edu.umh.es/query-phenoleaf-db/

2 http://bar.utoronto.ca/efp/cgi-bin/efpWeb.cgi

3 http://www.r-project.org/
