# Supplementary material for: A Network-Guided Genetic Approach to Identify Novel Regulators of Adventitious Root Formation in Arabidopsis thaliana
Source: Front Plant Sci. 2019 Apr 12;10:461. doi: 10.3389/fpls.2019.00461 (PMC6478000; doi:10.3389/fpls.2019.00461)
Supplement: Supplementary file 3 [file Data_Sheet_1.PDF]

A Network-Guided Genetic Approach to Identify Novel Regulators of Adventitious Root Formation in *Arabidopsis thaliana*

Sergio Ibáñez, Helena Ruíz-Cano, María Ángeles Fernández, Ana Belén Sánchez-García, Joan Villanova, José Luis Micol, José Manuel Pérez-Pérez*

*** Correspondence:** José Manuel Pérez-Pérez (jmperez@umh.es)

1 Supplementary Figures and Tables

1.1 Supplementary Figures

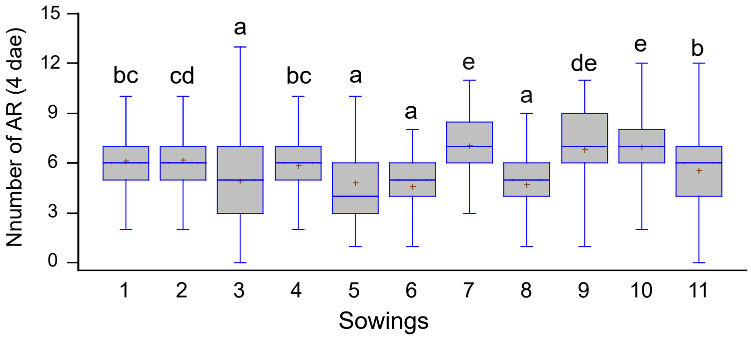

Figure S1.- Box-plots of adventitious root number in Col-0 hypocotyls according to the different sowings. Letters indicate statistically significant differences (LSD; p-value<0.05) between sowings. dae: days after excision.

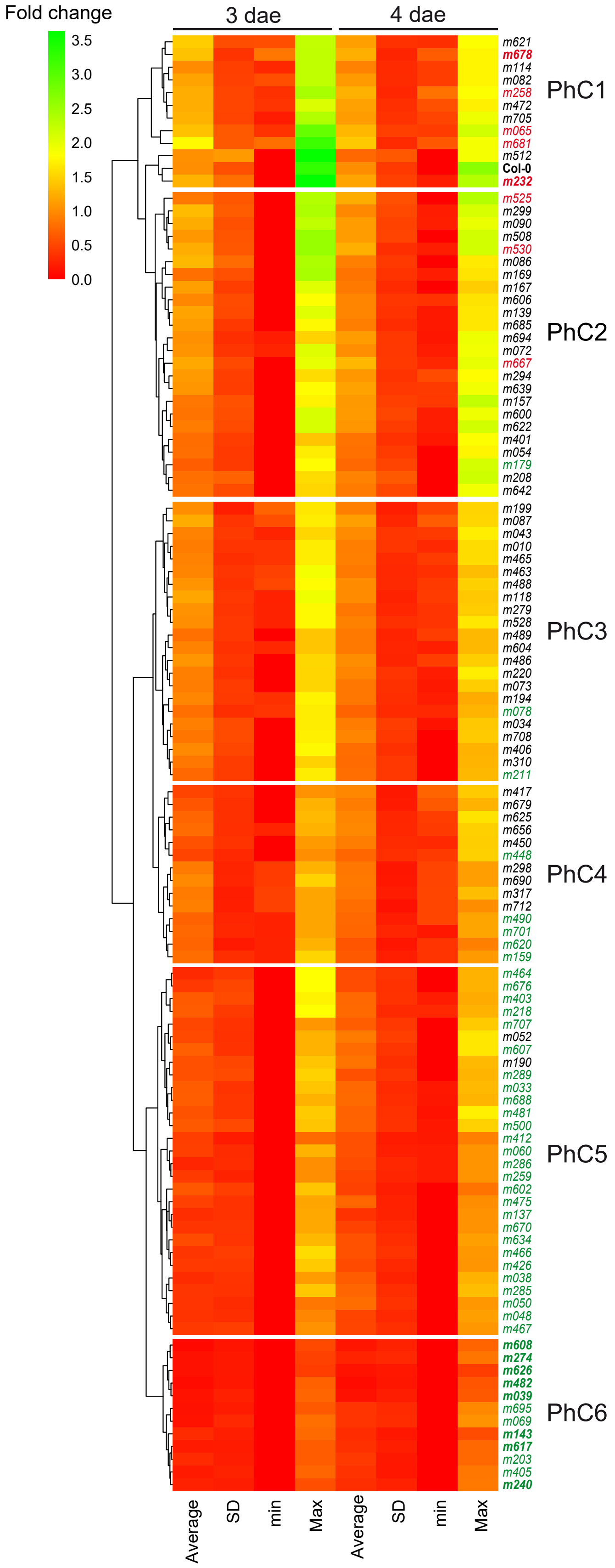

Figure S2. Heat map representation of relative rooting capacity in the studied lines. Each row corresponds to a single mutant, and the color scale corresponds to the fold change of rooting capacity values relative to the Col-0 background. Manhattan distance matrix between lines as regards their AR formation relative values was calculated to build the dendrogram. Six phenotypic clusters, PhC1 to PhC6, were defined based on this dendrogram. Mutant lines with significant changes over Col-0 are shown in green (lower AR values) or red (higher AR values), and mutant lines selected for further studies are indicated in bold; dae: days after excision.

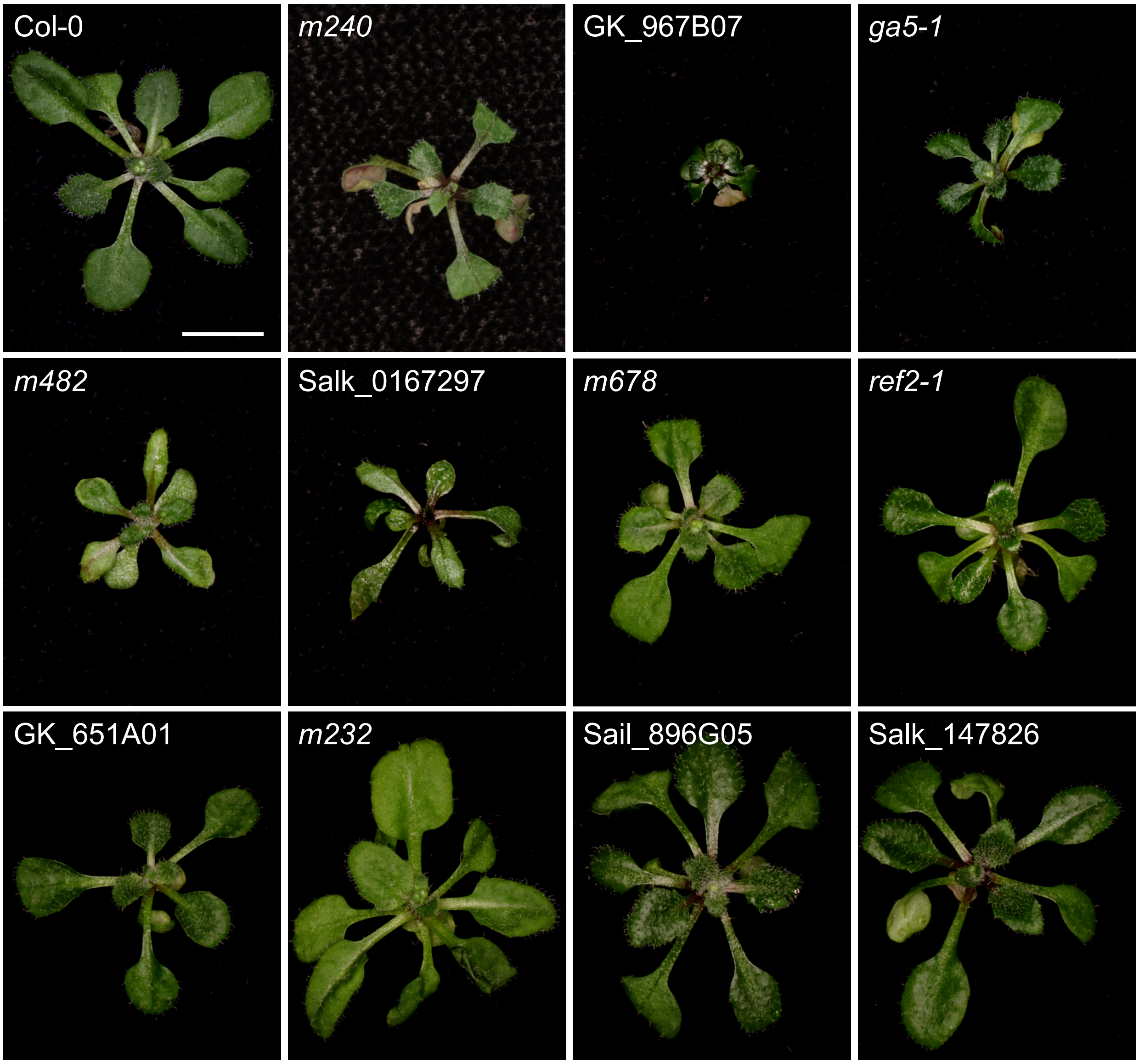

Figure S3. Rosettes of the *lars* and *mars* mutants studied in this work. Pictures were taken 21 d after sowing in Petri dishes. Scale bar: 5 mm.

1.2 Supplementary Tables

Table S1. Relative gene expression data of the 339 expressed genes with confirmed homozygous T-DNA insertions in the PhenoLeaf collection. The expression value of a given gene was calibrated relative to the mock expression in each experiment by a Log_2 transformation. CIM: callus induction medium; cot: cotyledon explants; pet: petal explants; RIM: root induction medium; SIM: shoot-induction medium.

Table S2. AR number variation in the hypocotyl after whole root excision in the studied lines. The T-DNA homozygous lines displaying significant differences (LSD; p-value<0.05) in AR number as regards Col-0 at 3 and 4 days after excision were selected for further studies.
